# “‘Oh gosh, why go?’ cause they are going to look at me and not hire”: intersectional experiences of black women navigating employment during pregnancy and parenting

**DOI:** 10.1186/s12884-022-05268-9

**Published:** 2023-01-10

**Authors:** Renee Mehra, Amy Alspaugh, Jennifer T Dunn, Linda S Franck, Monica R McLemore, Danya E Keene, Trace S Kershaw, Jeannette R Ickovics

**Affiliations:** 1 Department of Chronic Disease Epidemiology, Yale School of Public Health, 60 College St, CT 06520 New Haven, USA; 2 Department of Family Health Care Nursing, School of Nursing, University of California, San Francisco, UCSF Box 0606, CA 94143 San Francisco, USA; 3 College of Nursing, University of Tennessee, 1200 Volunteer Blvd, Knoxville, 37916 USA; 4 Department of Social and Behavioral Sciences, School of Nursing, University of California, 490 Illinois Street, CA San Francisco, 94158 USA; 5 Department of Social and Behavioral Sciences, Yale School of Public Health, 60 College St, CT 06520 New Haven, USA

**Keywords:** Employment, Financial insecurity, Income inequality, Intersectionality, Mental health, Perinatal health, Pregnancy discrimination, Qualitative research, Stress

## Abstract

**Background:**

Workplace legal protections are important for perinatal health outcomes. Black birthing people are disproportionally affected by pregnancy discrimination and bias in the employment context and lack of family-friendly workplace policies, which may hinder their participation in the labor force and lead to gender and racial inequities in income and health. We aimed to explore Black pregnant women’s experiences of pregnancy discrimination and bias when looking for work, working while pregnant, and returning to work postpartum. Additionally, we explored Black pregnant women’s perspectives on how these experiences may influence their health.

**Methods:**

Using an intersectional framework, where oppression is based on intersecting social identities such as race, gender, pregnancy, and socioeconomic status, we conducted an analysis of qualitative data collected for a study exploring the lived experience of pregnancy among Black pregnant women in New Haven, Connecticut, United States. Twenty-four women participated in semi-structured interviews (January 2017-August 2018). Interview transcripts were analyzed using grounded theory techniques.

**Results:**

Participants expressed their desire to provide a financially secure future for their family. However, many described how pregnancy discrimination and bias made it difficult to find or keep a job during pregnancy. The following three themes were identified: 1) “You’re a liability”; difficulty seeking employment during pregnancy; 2) “This is not working”; experiences on the job and navigating leave and accommodations while pregnant and parenting; and 3) “It’s really depressing. I wanna work”; the stressors of experiencing pregnancy discrimination and bias.

**Conclusion:**

Black pregnant women in this study anticipated and experienced pregnancy discrimination and bias, which influenced financial burden and stress. We used an intersectional framework in this study which allowed us to more fully examine how racism and economic marginalization contribute to the lived experience of Black birthing people. Promoting health equity and gender parity means addressing pregnancy discrimination and bias and the lack of family-friendly workplace policies and the harm they cause to individuals, families, and communities, particularly those of color, throughout the United States.

**Supplementary Information:**

The online version contains supplementary material available at 10.1186/s12884-022-05268-9.

## Background

Over the past 100 years there was a large increase in women in the United States (US) labor force such that women comprised 47.4% of the total labor force in 2019 compared to 20.3% in 1920 [1–3]. Despite the overall reduction in the US labor force during the severe acute respiratory syndrome coronavirus 2 (SARS-CoV-2), also known as coronavirus disease 2019 (COVID-19) pandemic, women still comprised 47.0% of the total labor force in 2020 [3, 4]. Two out of three women who are pregnant in the US work during pregnancy [5] and most continue working until, on average, three weeks until their due date [6]. Nearly one in four women return to work within two weeks of giving birth [7], with almost three quarters of women reporting that the primary reason for returning to work was the inability to afford taking off more time [6]. Policies that protect women in the workplace, particularly during pregnancy and the postpartum period, are important for improving maternal and infant health outcomes and workplace conditions [8]. We acknowledge that not all people with the capacity for pregnancy identify as women, therefore, we use gender-inclusive language unless gender-specific language was used in cited publications.

US workplace legal protections against pregnancy discrimination in the employment context include the Pregnancy Discrimination Act (see Table 1). Under this Act, an employer cannot discriminate against women with respect to hiring or other conditions of employment, including pay, fringe benefits, promotions, job assignments, training, layoffs, and firing, because of a pregnancy-related condition, if they are able to perform the major functions of their job [9]. A review of extant research indicates that despite laws prohibiting pregnancy discrimination, reports from government commissions and non-government organizations indicate that women experience both informal and formal forms of this discrimination [8]. Between 2010 and 2015, almost 31,000 complaints of pregnancy discrimination were filed in the US [10]. These complaints have increased over time, and women of color, particularly Black women, are disproportionately impacted [11, 12]. However, little is known about pregnancy discrimination and its effects on maternal and infant health or how birthing people experience this discrimination, particularly Black pregnant and birthing people who are most likely to be impacted by this type of discrimination.

**Table 1:** The Pregnancy Discrimination Act

•The Pregnancy Discrimination Act, was enacted in 1978 to prohibit sex discrimination “because of or on the basis of pregnancy, childbirth, or related medical conditions; and women affected by pregnancy, childbirth, or related medical conditions shall be treated the same for all employment-related purposes, including receipt of benefits under fringe benefit programs, as other persons not so affected but similar in their ability or inability to work.” [9].
•The Pregnancy Discrimination Act makes it illegal, among other things, to not hire a person because of a current or future pregnancy, fire or demote a pregnant employee, deny the same or similar job to an employee when returning from pregnancy-related leave, treat a pregnant employee differently than other temporarily disabled employees by refusing reasonable accommodations, or harass a pregnant employee related to their pregnancy [9].
•Employers are required to treat a woman who is temporarily unable to perform her job due to pregnancy the same as other temporarily disabled employees, by providing accommodations such as light or modified tasks, alternative assignments, disability leave, and leave without pay [8, 9]. The labeling of pregnancy-related conditions as a "disability" is the subject of ongoing debate and cross-disciplinary advocacy [13]. However, the current legal mechanism for asserting rights to reasonable accommodation during pregnancy in the US is through a disability framework [14].

A study of employed, predominately white, pregnant women found that perceived pregnancy discrimination was associated with increased perceived stress, which was in turn associated with increased postpartum depressive symptoms and decreased gestational age, birth weight, and number of pediatric visits [15]. Employed, predominately white, pregnant women who anticipated pregnancy discrimination were more likely to conceal a pregnancy, which was in itself associated with higher anxiety and depression [16]. A national survey in the US found that most women who worked while pregnant needed and asked for workplace accommodations, and that their employers generally attempted to provide such accommodations [6]. However, a recent qualitative study among employed pregnant Black and white women at risk of preterm birth in Durham, North Carolina, found that women experienced pregnancy discrimination through involuntary reduction in hours, refusal to provide workplace accommodations, and termination when they requested work accommodations recommended by their healthcare provider [17].

Additional workplace legal protections for birthing people include parental and medical leave during pregnancy or after the birth of an infant. The Family and Medical Leave Act (FMLA) provides up to 12 weeks of unpaid, job-protected leave for eligible employees working for public agencies and private sector employers with 50 or more employees [18]. Unpaid family leave is only available to 88% of civilian workers in the US [19]. However, due to pre-existing economic disparities, Black birthing people are more likely to not take family and medical leave because of financial constraints or have difficulty making ends meet while on family and medical leave [20]. Moreover, among high-income countries, the US is alone in failing to provide universal paid parental and family leave [21]. Until recently, US policy has focused on employer mandates and corporate tax incentives to increase access to parental and family leave; a policy approach that has exacerbated disparities in access to benefits and leave [22]. Only 14–40% of workers have access to paid family leave, less than 8% of lowest wage earners have access to paid leave, and Black and Latine workers are less likely to have access to paid parental or family leave compared to white workers [19, 22]. Prior research found that the benefits of paid family leave include higher likelihood of initiation and longer duration of breastfeeding [23], increases in hours worked and income of employed mothers of 1- to 3-year-old children [24], and decreases in postneonatal mortality rates [25]. However, one qualitative study found that parents’ decision making around paid family leave is limited by lack of information, guidance, and benefits [26].

Pregnancy discrimination is common and complaints regarding pregnancy discrimination have increased, particularly among Black women and women of color. Additionally, the lack of family-friendly workplace policies may disproportionally affect Black birthing people due to social and economic structures that influence their employment. Yet, there is limited knowledge of Black birthing people’s experiences of discrimination and bias in the employment context (incorporating legally actionable discrimination and implicit or unconscious bias that impacts the employee’s experience in the workplace) and how these experiences may influence maternal and infant health and racial disparities in maternal and infant health. Furthermore, much of the existing research on pregnancy discrimination focuses on a single axis of social identity (i.e., pregnancy but not race) [16, 27]. To address these important gaps in the literature, we conducted a qualitative analysis of interviews with Black birthing people to better understand the intersectional aspects of their lived experience of looking for work, working while pregnant, and returning to work postpartum. We also explored birthing people’s perspectives on how these experiences may influence their health.

We used an intersectional framework to examine experiences of discrimination and bias in the employment context along the multiple axes of race, gender, pregnancy, and socioeconomic status and how these social identities interact to construct and reinforce systems of power and oppression. Legal scholar Kimberlé Crenshaw first introduced the term “intersectionality” in 1989 to describe the multiple axes of oppression experienced by Black women based on the relationship between their gender and their race [28, 29]. Intersectionality theory and practice has expanded from its original focus on race and gender to include additional identities and oppressions, such as age, socioeconomic status, ability, sexual orientation, national origin, and immigration status [30]. Although there is power in describing how each of these axes operates separately to oppress Black birthing people, the authors take an intersectional approach to examine how these social identities mutually constitute and shape the lived experience of the participants. For a further explanation of intersectionality in the workplace, see Table 2.

**Table 2:** Intersectionality in the workplace

Crenshaw frequently uses the example of DeGraffenreid v. General Motors to illustrate the way in which the application of a “single axis” framework fails to account for ways in which multiple axes of one’s social identity interact to constitute and reinforce discrimination and oppression. In Degraffenreid, Black women plaintiffs brought a claim of employment discrimination against General Motors, alleging that the exclusion of Black women from employment in the company was the result of compounded discrimination based on their race and gender. The court dismissed the plaintiffs’ case, finding that plaintiffs did not state a valid gender discrimination claim because General Motors employed white women in office jobs and further that plaintiffs failed to state a legally cognizable race discrimination claim because the company employed Black men in factory jobs (Crenshaw 1989).
In our research, we understand the intersectionality of our participants in the following way: 1. First axis of oppression: racial bias and discrimination (racism) 2. Second axis of oppression: sex/gender bias and discrimination 3. Third axis of oppression: pregnancy bias and discrimination 4. Fourth axis of oppression: socioeconomic status bias and discrimination

## Methods

### Design and setting

This is an analysis of qualitative data collected as part of a study exploring neighborhood-level structural opportunities and barriers, health behaviors, stress, and experiences of discrimination among Black pregnant women in New Haven, Connecticut. We previously identified multiple forms of intersectional discrimination that participants experienced in everyday contexts [31]. This analysis focused on neighborhood access to employment opportunities and the dominant emerging theme of pregnancy discrimination and bias in employment-related contexts. Specifically, in this analysis, we sought to understand the intersectional experience of Black pregnant women in looking for work, working while pregnant, and returning to work postpartum. Participants referred to themselves as women and mothers, accordingly in our research findings we used these same terms.

Study methods are described in detail elsewhere [31]. Study details are reported using the Consolidated criteria for reporting qualitative research (COREQ) [32]. Eligibility criteria included pregnant women who were at least 18 years of age and self-identified as African American or Black. We aimed to diversify our sample based on socioeconomic status, as experiences of discrimination may differ by this characteristic [33]. We characterized socioeconomic status by primary source of financial support, that is public assistance benefits or employment (i.e., the participant, their partner, or a family member was employed). Participants had no prior relationship or knowledge of the researchers. We recruited participants via flyers (see Additional file 1) in the community that indicated that the aim of the study was to better understand the effects of neighborhoods on pregnancy and that the study would explore things that pregnant women do to have a healthy pregnancy and the challenges they face.

After obtaining verbal informed consent from participants, author 1 conducted one-time, individual, in-person, semi-structured interviews using an interview guide iteratively developed by the researchers. Interview questions that elicited responses relevant to this analysis on pregnancy discrimination and bias included: Can you tell me what this pregnancy has been like for you? Are there things that are stressful (or make you worry) during your pregnancy? Can you tell me about opportunities to work in your neighborhood? How does your neighborhood help you have a healthy pregnancy? Interviews were 45 to 60 min in length and were conducted at the Yale School of Public Health between January 2017 and August 2018. Only the participant and interviewer were present at the interview. Participants received $40 for their time and involvement. Interviews were recorded and transcribed verbatim. Transcripts were not returned to participants for comment. Ethical approval was received from Yale University Institutional Review Board (Human Subjects Committee Protocol ID number: 1611018675).

### Analysis

Three trained researchers used an iterative and inductive coding approach adapted from grounded theory [34]. Codes and themes were derived from the data and interview guide. Coding reliability was established by iteratively choosing small samples of interviews for open coding and double coding. Coding issues were resolved through consensus. Author 1 applied the final codebook to all transcripts using ATLAS.ti software (Version 8, Scientific Software Development GmhH, Germany, 2018). Data saturation was achieved for the original study. Author 1 wrote field notes after conducting interviews to assess data saturation and provide contextual details for data interpretation, and memos during data interpretation to monitor biases. Codes related to discrimination in the employment context were used for this analysis. Authors 1 and 2 read transcripts to contextualize these codes within participants’ broader narratives. Participants did not provide feedback on findings.

### Description of author’s backgrounds

All authors are trained and/or experienced in qualitative research methodology. Author 1, an Asian and white female, was a doctoral candidate at the time of conducting the interviews and a postdoctoral scholar at the time of analyzing and interpreting the data for this analysis. Author 2 is a white female assistant professor at a College of Nursing. Author 3 is a white female lawyer with expertise in reproductive health, civil rights, and employment law. Author 4 is a white female professor of nursing. Author 5 is a Black female associate professor of nursing. Author 6 is a white female associate professor of public health. Author 7, a white male, and Author 8, a white female, are professors in the social and behavioral sciences department of a school of public health. The authors, who are all fully employed in or affiliated with academic institutions, bring their own personal experiences and understanding of pregnancy discrimination and bias to this work, which may affect their interpretation of the data. Specifically, in analyzing these data, authors bring their lived experience of pregnancy discrimination and bias and various types of employment experiences, including tip-based food services, and the benefits and flexibility that are or are not provided to employees in these types of employment.

## Results

### Study sample

Twenty-four women (aged 21 to 45 years, median of 32 years) participated in the study. No participants refused to participate or dropped out of the study. The gestational age of participant’s pregnancies ranged from 5 to 38 weeks (median of 22.5 weeks). Fourteen participants (58%) were pregnant for the first time. The remaining participants reported having between 1 and 5 children prior to the current pregnancy. For 14 participants, the primary source of financial support at the time of the interview was employment (i.e., the participant, their partner, or a family member was employed); for the remaining 10 participants, the primary source was public assistance benefits. Participants were working or had worked in the following job sectors: retail, education (including universities), healthcare, and government. The topic of pregnancy discrimination and bias in the employment setting was present in *n* = 13; 54.2% of interviews.

### Findings

**Fig. 1:**
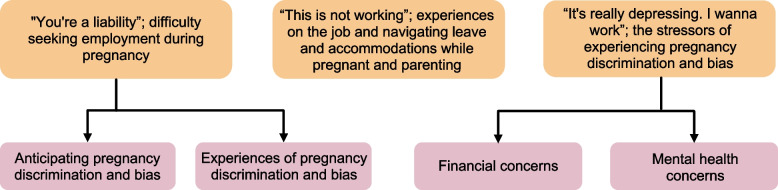
Thematic map of pregnancy discrimination and bias in the employment context

Participants in our study expressed a desire to “be independent and provide for my child” (first-time pregnant woman in her early 20s who is supported by her family), by giving their child “the best life and opportunities possible” (first-time pregnant, married woman in her early 30s who is employed), and in many cases to provide a life for their infant that improved upon their own. To this end, participants desired to have a job and be financially secure. However, many participants, regardless of their socioeconomic status, reported having difficulty finding or keeping a job both during and after current or prior pregnancies as a result of pregnancy discrimination and bias in the employment context. The following themes were identified (Fig. 1): 1) “You’re a liability”; difficulty seeking employment during pregnancy; 2) “This is not working”; experiences on the job and navigating leave and accommodations while pregnant and parenting; and 3) “It’s really depressing. I wanna work”; the stressors of experiencing pregnancy discrimination and bias. Overall, participants in this study were either experiencing, or planning around, pregnancy discrimination and bias and lack of family-friendly workplace policies throughout their reproductive years in a way that caused immense financial burden and stress.

### **“You’re a liability”; difficulty seeking employment during pregnancy**

Difficulty seeking employment during pregnancy was common for participants in this study and affected both those who were unemployed and looking for work and those who were already employed but desiring to change jobs. Many participants discussed their feelings about anticipating discrimination while looking for work, and a few participants discussed experiencing discrimination while on the job market.

#### Anticipating pregnancy discrimination and bias

Many participants spoke about the anticipation of pregnancy discrimination and bias while seeking employment. This anticipation of pregnancy discrimination and bias dissuaded some participants from applying for jobs or following up with interviews when they were offered, because they believed that a known pregnancy would render their efforts futile. Participating in job interviews while visibly pregnant was especially challenging. One participant discussed that finding a job was a much more difficult and greater source of stress during a pregnancy than it normally was, especially since her pregnancy had just become more visibly obvious. She said: “I’ve looked online and made appointments, but then I tell myself, ‘Oh gosh, why go?’ Cause they are going to look at me and not hire.” (Mother of 5, single, in her early 40s who is a public assistance recipient.) The visibility of her pregnancy kept her from attending job interviews, despite her need for income. Similarly, many participants were not optimistic about gaining employment while pregnant. One woman had applied for several jobs before becoming pregnant, but the interviews occurred after she became pregnant. She described avoiding any mention of her obvious pregnancy during the interview. However, she felt that her pregnancy was the reason she was not ultimately hired: “[The interviewers] never said anything about the pregnancy. I didn’t mention it in any of the paperwork or anything, umm but I was showing. So, I pretty much think that was the reason.” (First-time pregnant woman in her early-30s who is supported in part by the father.) After three unsuccessful interviews, she concluded that her visible pregnancy was impeding her employment options.

Participants understood that potential employers would view their pregnancy, and by extension the participants themselves, as liabilities. One participant explained this unstated discrimination: “Because a lot of employers don’t want to hire you because you’re a liability to the company. If something happens to you while you’re pregnant on the job, their kinda liable for it.” (First-time pregnant, married woman in her mid-20s who is a public assistance recipient.) While it may have manifested differently for each participant, the understanding that being visibly pregnant was a liability while seeking employment was a common experience for participants in this study.

#### Experiences of pregnancy discrimination and bias

While less common, several participants discussed specific instances in which they were told directly by the employer that their pregnancy would prevent them from getting a job. The language of liability was used, with the emphasis placed on the legal and economic security of the organization instead of centered on the health of the woman and her fetus. One participant described her experience of a job offer being revoked because of her pregnancy: “[I was] offered a position and then I let them know that I was pregnant and just like, ‘Ah well, I’ll call back’, you know, kind of run around. I kinda know what’s going on.” (First-time pregnant, married woman in her mid-20s who is a public assistance recipient.) Another participant was given weight-bearing restrictions by her healthcare provider due to threatened preterm labor. At the time, she worked as a certified nursing assistant. She was then told by her agency that they could not place her in a position because of her pregnancy: “I was looking for jobs, going to job interviews, but they wouldn’t hire me because I’m pregnant… They said I’m a liability to the company and they said I could like injure myself, I could slip and fall, whatever and I could sue them, and they don’t want that. So, like, that’s not fair, there’s a lot of pregnant women that work. So, they think you are a liability, you’re just too far along.” (First-time pregnant woman in her early 20s who is supported by her family.)

Participants employed in jobs that required a significant amount of manual labor, as noted above, were more likely to describe experiences of pregnancy discrimination and bias. Another participant who worked as a certified nursing assistant had her ability to physically do her job questioned: “And then when I go to some jobs cause I was working through an agency, they’re like, ‘She’s big, well how much could she do?’” (Mother of two in her early 30s who is supported by her partner.) This participant wanted to continue working in her job but faced an employment agency that was unwilling to accommodate potential temporary work limitations related to her pregnancy. When the agency provided no safe alternatives, the participant and her employer jointly made the decision that she should not work for them.

### **“This is not working”;experiences on the job and navigating leave and accommodations while pregnant and parenting**

Pregnancy discrimination and bias did not just affect participants seeking employment, it also affected how supported they felt in their job and their desire to remain or change jobs in the future, regardless of job sector. One participant discussed how, since her first and second pregnancy, she has been treated drastically different at work. She described the weekly, if not daily, scrutiny she received at work, saying: “I’m making deadlines and doing everything else, but I’m treated kind of as an outsider, or just not a part of the team, or different notably different. So, I don’t know what the contributing factors could be. So, one could be race because I am the only person [of color]. One could be pregnancy.” She described that after having her first child, her work environment had become more racially charged, which was on top of the existing intense and demoralizing situation she felt being judged by her co-workers on the basis of race: “You are judged on your intelligence because of your skin color. You’re judged on your competence because of your skin color.” After becoming pregnant for the second time, she perceived that her co-workers questioned her competence and performance: “[Co-workers] don’t trust that you know what you’re doing or that you’re paying enough attention to detail.” (Mother of one, married, in her mid-30s who is employed.) This scrutiny and stress motivated her to leave a work environment she described as toxic and look for a new job.

Another participant discussed an experience with a prior job not giving her appropriate time off after an emergency operative birth: “I was actually trying to be very cognizant of when we started trying and like when the baby would be due to not put a strain on the department, which in hindsight it sounds awful, but I should not have cared, because they clearly didn’t show me the same respect that I was trying to show to them.” (Mother of one, married, in her early 30s who is employed.) This lack of mutual consideration led her to prioritize finding another job with better benefits and flexibility before having more children. The new job with better benefits and a supportive manager enabled a better work and pregnancy experience with her second child.

Disclosing one’s pregnancy status at work was also a source of stress for many participants early in their pregnancy. The perception of getting special considerations for their pregnancy status, getting unwanted attention, or experiencing harassment made participants more likely to delay the disclosure of their pregnancy to their supervisor or co-workers. One participant, pregnant with her first child, said: “I don’t want nobody to try to feel sorry for me like, you know, I’m pregnant. Oh, you can’t do this and you can’t do that type of thing. And I don’t want that to be, you know, the front line or like I’m trying to take advantage somehow. So, I want to be able to keep it confidential right now with myself before telling them, so that way it’s not like I’m using the pregnancy as, you know, something.” (First-time pregnant woman, single, in her early 20s who is employed.) For this participant, fear of discrimination centered around poor treatment from her co-workers.

Participants also had to manage the birth of their infant within the context of work and family constraints, and this sometimes led to difficult choices that could affect their own health. Participants described balancing their desire to bond with their infant with their desire or need to return to work because of limited family and medical leave policies, with an important deciding factor being the availability of social support. It was so important for one participant to spend more time with her newborn that she planned to have a repeat operative birth due to the longer medical leave associated with an operative birth: “And I just think honestly, I don’t think you get enough time to, you know, six weeks home with the baby, that’s kinda short to me. So, I assume [if] I take a c-section I’m going to be out longer, and I don’t mind… If I could get just a little extra time like an extra month that would mean so much to be home with my baby.” (Mother of one in her early 30s who is employed.) With limited workplace and social support, this participant made medical decisions based not on her own health concerns and priorities but based on getting minimally sufficient time to recover and bond with her infant.

### **“It’s really depressing. I wanna work”; the stressors of experiencing pregnancy discrimination and bias**

For participants in this study, experiences with pregnancy discrimination and bias manifested in many different areas of their life. Because employment is tied not only to income, but also to health insurance and other health benefits, difficulty finding a job or concerns regarding a job during or after pregnancy could be a major life stressor. In particular, participants reported stress related to the financial concerns of pregnancy discrimination and bias. These stressors, in turn, could create or exacerbate mental health issues.

#### Financial concerns

Participants reported financial stress from pregnancy discrimination and bias regardless of socioeconomic position. However, financial stress was most pronounced for participants who were single or unpartnered. One participant, who was pregnant with her second child, was in the midst of filing for divorce. Additionally, poor treatment perceived as potentially pregnancy and racial discrimination in her current job necessitated her looking for a new job. She explained her situation, saying: “I’m looking for a new job… I take the emotion out of it because it’s, if I didn’t have self-confidence, it would make you cry, it would make you feel like OK what is wrong with me or what am I not doing because I have so much other stuff on my plate. I just can’t afford to expend emotional energy in that area because I’m just balancing and trying to provide for my family. So, it’s like OK if this is not working. I need to find something that will work and continue to perform to the best of my ability but continue to look for something else.” (Mother of one, married, in her mid-30s who is employed.) For this participant, the perceived experience of pregnancy discrimination and bias added to her personal and financial concerns.

A participant who was not given a job after disclosing her pregnancy status reported financial stress at not having the income, but acknowledged: “You know financially it is quite, you know, nerve wracking a little bit, but with the help and support from my husband, I’m maintaining. I’m blessed for what I do have.” (First-time pregnant, married woman in her mid-20s who is a public assistance recipient.) Emotional and financial support from a romantic partner helped ease the stress of job instability during pregnancy. This may not be the experience with other social supports, however. One participant, who was being financially supported by her sister but was unable to work in her job due to pregnancy discrimination and bias, was very worried about her financial support. She wanted to keep her certified nursing assistant job, despite the agency’s concern about her pregnancy, because: “I need the money, I don’t want to depend on my sister because she’s done a lot for me already. I’m trying to be independent.” (First-time pregnant woman in her early 20s who is supported by her family.) She was upset with her inability to support her family and buy necessities, like a crib and baby clothes, that she knew her infant would need, saying: “I can’t do nothing for my child, that hurts me.” Different types of social support, and the absence of support, created different experiences for participants in this study, although some level of financial stress was noted among all three of these participants.

#### Mental health concerns

For many participants, concerns over their financial security were not only stressful but could also affect their mental health. Participants reported feeling a variety of negative emotions, such as feeling depressed that they could not find a job despite wanting to work, feeling angry and frustrated about not being able to work, and feeling disrespected and offended when they were told by potential employers that they could not do the work. One participant discussed an especially difficult day in which she considered ending her own life. When asked what was going through her mind when she was at her lowest, she said: “[It] probably was the whole job thing. It probably was the whole income thing and me just being depressed about that.” (First-time pregnant woman in her early-30s who is supported in part by the father.) While she received some help from the father, she felt unable to gain control of her circumstances in a way that would allow her to support her growing family.

Many participants spoke of trying to minimize stress because of its possible effects on their pregnancies and infants. A participant in her early 40s who is a public assistance recipient was specifically trying to minimize stress in an effort to avoid gestational hypertension and preterm birth that she experienced with other pregnancies. She was, however, looking for employment while pregnant and found this process very stressful. The participants in this study were keenly aware that despite their best intentions and positive health choices, the stress related to financial concerns compounded by pregnancy discrimination and bias could adversely affect their health during pregnancy and the health of their infant.

## Discussion

This analysis provides deeper insight of the lived experience of discrimination and bias in the employment context by Black pregnant women in the US. For the participants in our study, pregnancy discrimination and bias while seeking employment were both anticipated and experienced. Difficulty balancing the demands of pregnancy and parenting in the workplace affected their satisfaction in their current jobs and limited pursuit of new jobs. While demands and stress are commonplace for many expecting parents, the additional burden of pregnancy discrimination and bias for Black pregnant women within the workplace added stress, compounding financial and mental health concerns.

Many of our findings are supported by extant literature. A recent qualitative study examined how women at risk of preterm birth balanced work and pregnancy considerations. Although some women reported positive experiences regarding work accommodations, many also noted involuntary reduction in hours, truncated advancement, and termination of employment [17]. Similar to women in our study, having a job that allowed for flexibility regarding prenatal considerations and postpartum recovery was viewed positively by the study participants. Our study suggests that these considerations are not unique to those at risk for preterm birth, but instead speak to a more common discourse of the difficulty of working, particularly in lower-wage jobs, while pregnant and parenting.

Our findings regarding study participants’ mental health concerns related to anticipated or experiences of pregnancy discrimination and bias are consistent with other studies. Jones [16] found that anticipated pregnancy discrimination shaped women’s pregnancy disclosure behavior, which was associated with increasing levels of psychological distress. Moreover, Hackney and colleagues [27] found that perceived pregnancy discrimination was associated with an increase in maternal stress and postpartum depressive symptoms, as well as poorer birth outcomes. Stress, especially when associated with personal finances, has been shown to increase the incidence of postpartum depression [35, 36]. Maternal depression, especially in the postpartum period, has short and long-term deleterious physical and mental health consequences for both the birthing individual and the infant [37]. The effects of pregnancy discrimination and bias and lost wages have immediate and far-reaching ramifications for those who experience them.

Our qualitative findings add to existing quantitative research. First, our findings suggest that anticipation of pregnancy discrimination and bias is an important influence on Black birthing people’s lived experience in addition to experiences of pregnancy discrimination and bias in the workplace. Anticipation of discrimination may prevent people from even applying or interviewing for jobs. This limits the ability of people without work to find employment, and hinders job mobility, both upward and parallel, among those in the workforce. Secondly, our findings highlight how experiences of pregnancy discrimination and bias compound normal stressors of pregnancy, especially those regarding financial security. The financial burden of raising children causes stress among parents [38], but participants in our study reported that finding or changing jobs during pregnancy added to this financial strain. Using an intersectional framework allowed us to more fully examine how racism and economic marginalization contribute to the lived experience of stress experienced by Black birthing people, clearly exemplified by a participant in this study who shared that she wondered whether the aggression and poor treatment at work she experienced was due to her race or her pregnancy and subsequent parenthood. Ultimately, the multiple intersecting identities cannot be separated for marginalized and oppressed people and result in magnification of their experience of discrimination [30].

The financial stressors expressed by participants in our study are consistent with economic inequities that have long existed for Black people, especially Black women. A higher proportion of Black mothers work, compared to white, Asian, and Latina mothers [39, 40]. Additionally, Black women have higher labor force participation rates than white women across all levels of education [39, 40]. Higher labor force participation rates among Black women are attributed to factors such as the societal expectation of Black women’s employment as well as labor market discrimination against Black men leading to lower wages and less stable employment than white men [41], putting a greater burden on Black women to support their families. For example, a higher proportion of Black mothers (79%) are “breadwinners” (defined as earning at least 40% of a household’s income and earnings) compared to Latina (49%), white (48%), and Asian/Pacific Islander (43%) mothers [42]. However, due to a long history of discriminatory hiring practices and exclusionary workplace protections and public assistance policies, Black women are concentrated in low-paying, inflexible service occupations that are excluded from various worker protections and lack retirement benefits, health insurance coverage, and paid sick and maternity leave [41]. Black women in the US are paid, on average, 62 cents for every dollar earned by white men, equaling almost $100,000 in lost wages over the span of a 40-year career [43, 44]. The addition of children further exacerbates income inequity for women, who incur a 4% wage penalty for every child they have, or a 6% wage penalty for every child for those in low-wage jobs [45].

Although the data collection for this study occurred prior to the COVID-19 pandemic, the findings are that much more important given the current state of social and economic upheaval. The COVID-19 pandemic has taken an especially hard toll on the Black community. A disproportionate number of COVID-19 cases have occurred among Black people, and the death rate among Black people is almost double that of white people [46, 47]. Black women, in particular, are over-represented in frontline, essential jobs that carry a heavy health risk during a pandemic and are treated simultaneously as essential and expendable workers [48]. Black women are also over-represented in industries hardest hit by job losses, such as restaurants, retail, and hotels [43], with Black women facing the highest unemployment rate among Black and white men and women [49]. A recent study of the effects of the pandemic on pregnant women in Philadelphia found that Black pregnant women reported a greater likelihood of negative impacts on their employment, were more concerned about a lasting economic burden, and were more concerned about their prenatal, birthing, and postpartum experiences [50]. Research suggests the current pandemic will further exacerbate inequities in wealth creation in Black, Latine, and Indigenous communities, which will disproportionately affect Black women [51–53].

Although research on the experience of pregnancy discrimination is limited, there is a richer body of evidence exploring workplace discrimination. Discrimination in the workplace is a common experience for women, with Black, Latina, and Indigenous women experiencing significantly greater discrimination than white women [54]. Black people in the workplace are exposed to a unique form of racialized harassment that plays upon stereotypes of race and gender [55]. Discrimination in the workplace manifests as the absence of opportunities regarding career advancement, skill development, and difficulties in interracial interpersonal working relationships [56]. Furthermore, race-based discrimination in the workplace is a direct cause of increases in work-related stress [56].

### Strengths and limitations

One strength of this study is the inclusion of a socioeconomically diverse sample of participants, thus allowing us to explore experiences of pregnancy discrimination and bias in both women who were employed and unemployed. Furthermore, we recruited participants regardless of parity (number of live births) allowing us to explore a broader range of experiences from both pregnant and parenting women. While the overall purpose of the study was to explore experiences during pregnancy, we did not specifically set out to explore experiences of pregnancy discrimination and bias in the employment context, and we did not assess for data saturation with respect to this theme. Thus, a different framework or more targeted questions may have generated different results. Additionally, we collected limited information on the employment history of participants. Furthermore, racial discordance between the interviewer and participants may have influenced the type and depth of experiences shared by participants. We only enrolled Black pregnant women in New Haven, Connecticut, which at the time of the study had a higher level of unemployment (10.4%) than the country as a whole (6.6%) [57], so our findings may have differing transferability to other women and settings with lower unemployment.

### Implications

Based on the extant data and these findings, the authors propose a multidisciplinary approach to the issues identified by the Black pregnant and parenting women in this study.

#### Policy implications

Tying benefits to employment, especially with the uneven application of employer benefits across jobs and incomes, exacerbates race, sex, and income inequalities. This is most evident in who is and is not covered by employer-based benefits, such as paid parental leave and health insurance. The jobs with the lowest pay, least flexibility, and least protection are also those with the fewest family-friendly benefits; these jobs are disproportionately held by women of color. Because those who are already most economically marginalized are most likely to have jobs with no paid leave [58, 59], it is our recommendation that leave benefits be provided through state and federal programs without regard to the number of employees at the job site, duration of employment, or job classification. As politicians debate the future of paid family leave in the US, the extant research and these findings support federal legislation that: 1) guarantees universal paid leave for all employees, including hourly, part-time, self-employed, and contract workers; 2) is funded through the government, rather than through a patchwork of employer mandates and tax incentives that increases the race, gender, and economic divide; and 3) addresses any potential gaps in FMLA job protection for workers taking paid leave. These basic supports should be provided as a right or entitlement, unconnected to a particular job or type of employment. Other countries have achieved this support population-wide through universal healthcare and providing lengthy paid parental leave. Since Black mothers in the US are more likely to be employed in jobs with fewer benefits, compared to mothers of other races [42], ensuring that all jobs have sufficient benefits and protections may prevent pregnant people from enduring the limitations and stress of low-benefit jobs or having to find a new job with adequate benefits. The problems associated with health insurance and other benefits being attached to employment has been highlighted during the COVID-19 pandemic. This may be the time in our history where we have a momentum to change our practices at a systemic level.

#### Clinical implications

In instances where people require pregnancy accommodations from their employers, healthcare providers who write an appropriate work accommodation request can mean the difference between accommodations being met and an individual getting fired [60]. State-specific resources for letter writing can be found in Table 3. Although healthcare providers do not have the legal training to provide advice on employment matters, clinics and hospitals could provide information on pregnancy discrimination and leave produced by government agencies, such as the US Equal Employment Opportunity Commission, or a nonprofit legal organization. If a provider’s website includes educational materials for patients, they could also include links to employment information on their website. Referrals to social work, toll-free legal hotlines, and specific local resources could also be available in clinics who care for birthing people [61]. Screening for mental health conditions in pregnancy such as anxiety and depression should move beyond numerical scores and use open-ended follow-up questions to explore the possible root causes, especially in marginalized people, as participants in our study reported adverse mental health outcomes because of the financial and emotional stressors associated with pregnancy-related employment concerns.

**Table 3:** Additional resources for pregnant and parenting people and healthcare providers

Website	Description of resource
www.pregnantatwork.org	This online resource center provides tools and educational materials for pregnant and breastfeeding workers, the healthcare professionals who treat them, and the attorneys who represent them. It also has useful materials for companies, human resources professionals, and management attorneys that can assist in navigating the many legal and practical considerations around pregnancy and breastfeeding accommodations.
www.acog.org	Portfolio of resources and position statements from the American College of Obstetricians and Gynecologist regarding employment considerations for pregnancy, postpartum, and breast/chestfeeding
www.abetterbalance.org	A Better Balance uses the power of the law to advance justice for workers, so they can care for themselves and their loved ones without jeopardizing their economic security. Includes a national legal helpline at 1-833-NEED-ABB for free and confidential information about workplace rights.

#### Research implications

Despite existing legal protections, complaints regarding pregnancy discrimination have increased over the past several decades, driven largely by complaints filed by women of color [10, 11, 62]. Coupled with the findings from our analysis and others [17], it is evident that existing legislation is insufficient to protect people with the capacity for pregnancy from experiencing pregnancy discrimination and bias. More research is necessary to further explore the experiences of pregnancy discrimination and bias in the employment context and its effects on the health, wealth, and well-being of Black pregnant and birthing people in the US. An emphasis on exploring pregnancy discrimination and bias in the employment context, specifically within Black, Indigenous, People of Color (BIPOC) and lower-income communities, is critical to understanding what is and is not working within the workplace, and why. Deeper investigation into the system’s shortcomings (and successes) can equip advocates and policymakers with the information and data needed to advocate for state and federal policies that better meet the needs of people most impacted by pregnancy discrimination and bias.

## Conclusion

Despite legal protections, pregnancy discrimination and bias is pervasive in the US and is more likely to affect those with the least economic and social resources [63]. We found that Black people with the capacity for pregnancy experienced pregnancy discrimination and bias which was harmful to their economic prospects and financial wellbeing, job stability and satisfaction, and mental health. Promotion of health equity and gender parity means addressing pregnancy discrimination and bias in the employment context and the lack of family-friendly workplace policies and the harm they cause to individuals, families, and communities, particularly those of color, throughout the US.

## Supplementary Information

**Additional file 1.**

## Data Availability

The datasets used and/or analyzed during the current study are not publicly available due to the sensitive nature of the interviews.

## Abbreviations

BIPOC: Black, Indigenous, People of Color
COREQ: Consolidated criteria for reporting qualitative research
COVID-19: Coronavirus disease 2019
FMLA: Family and Medical Leave Act
SARS-CoV-2: Severe acute respiratory syndrome coronavirus 2
US: United States
