# Supplementary material for: “‘Oh gosh, why go?’ cause they are going to look at me and not hire”: intersectional experiences of black women navigating employment during pregnancy and parenting
Source: BMC Pregnancy Childbirth. 2023 Jan 10;23:17. doi: 10.1186/s12884-022-05268-9 (PMC9830615; doi:10.1186/s12884-022-05268-9)
Supplement: Supplementary file 1 — Additional file 1. [file 12884_2022_5268_MOESM1_ESM.pdf]

Are you pregnant?

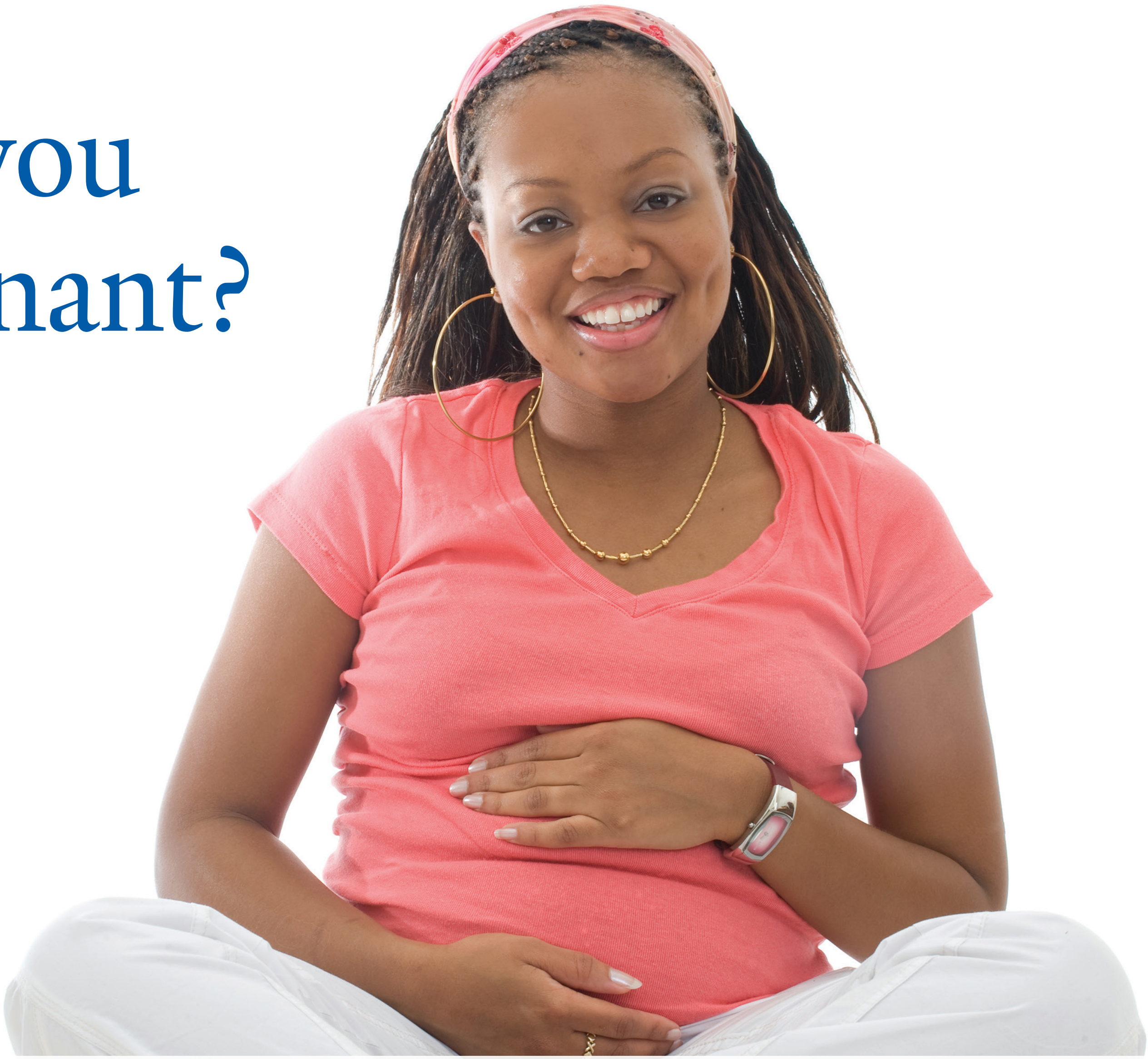

Pregnancy Study

If you are **18 years or older, pregnant, and African-American**, you may be eligible to participate in the Pregnancy Study. This study explores the things that women do to have a healthy pregnancy and the challenges they face. ***The interview will last 45-60 minutes and you will receive $40 for participating.***

To learn more or see if you are eligible to participate, contact x at (xxx) xxx-xxxx

The aim of this research study is to better understand the effects of neighborhoods on pregnancy among African-American women.

HSC #1611018675

HELP US DISCOVER | *Be Part of* Clinical Research at Yale.

Yale
